# Mitochondrial NAD(P)H *In vivo*: Identifying Natural Indicators of Oxidative Phosphorylation in the ^31^P Magnetic Resonance Spectrum

**DOI:** 10.3389/fphys.2016.00045

**Published:** 2016-03-30

**Authors:** Kevin E. Conley, Amir S. Ali, Brandon Flores, Sharon A. Jubrias, Eric G. Shankland

**Affiliations:** ^1^Department of Radiology, University of Washington Medical CenterSeattle, WA, USA; ^2^Department of Physiology and Biophysics, University of Washington Medical CenterSeattle, WA, USA; ^3^Department of Bioengineering, University of Washington Medical CenterSeattle, WA, USA

**Keywords:** magnetic resonance spectroscopy, ^31^P MRS, nicotinamide adenine dinucleotide, NAD^+^, NADP^+^, muscle, exercise

## Abstract

Natural indicators provide intrinsic probes of metabolism, biogenesis and oxidative protection. Nicotinamide adenine dinucleotide metabolites (NAD(P)) are one class of indicators that have roles as co-factors in oxidative phosphorylation, glycolysis, and anti-oxidant protection, as well as signaling in the mitochondrial biogenesis pathway. These many roles are made possible by the distinct redox states (NAD(P)^+^ and NAD(P)H), which are compartmentalized between cytosol and mitochondria. Here we provide evidence for detection of NAD(P)^+^ and NAD(P)H in separate mitochondrial and cytosol pools *in vivo* in human tissue by phosphorus magnetic resonance spectroscopy (^31^P MRS). These NAD(P) pools are identified by chemical standards (NAD^+^, NADP^+^, and NADH) and by physiological tests. A unique resonance reflecting mitochondrial NAD(P)H is revealed by the changes elicited by elevation of mitochondrial oxidation. The decline of NAD(P)H with oxidation is matched by a stoichiometric rise in the NAD(P)^+^ peak. This unique resonance also provides a measure of the improvement in mitochondrial oxidation that parallels the greater phosphorylation found after exercise training in these elderly subjects. The implication is that the dynamics of the mitochondrial NAD(P)H peak provides an intrinsic probe of the reversal of mitochondrial dysfunction in elderly muscle. Thus, non-invasive detection of NAD(P)^+^ and NAD(P)H in cytosol vs. mitochondria yields natural indicators of redox compartmentalization and sensitive intrinsic probes of the improvement of mitochondrial function with an intervention in human tissues *in vivo*. These natural indicators hold the promise of providing mechanistic insight into metabolism and mitochondrial function *in vivo* in a range of tissues in health, disease and with treatment.

## Introduction

Nicotinamide adenine dinucleotide is a co-enzyme that is integral to cell and mitochondrial metabolism (Williamson et al., 1967; Stubbs et al., 1972; Nicholls and Ferguson, 2002; Ying, 2008; White and Schenk, 2012) and has a signaling role in mitochondrial biogenesis (Imai, 2011; Canto and Auwerx, 2012). NADH is a key player in oxidative phosphorylation in mitochondria (Nicholls and Ferguson, 2002) and in glycolysis (Williamson et al., 1967; Stubbs et al., 1972), while the related metabolite, NADP, is important in anti-oxidant protection (Ying, 2008). Optical methods detect the sum of these compounds (NAD^+^ and NADP^+^ = NAD(P)^+^) and can resolve their cellular location to provide natural indicators of mitochondrial function and cytosolic redox state (NAD(P)H/NAD(P)^+^) (Jobsis and Duffield, 1967; Barlow and Chance, 1976; Wendt and Chapman, 1976; Scholz et al., 1995; Mayevsky and Rogatsky, 2007; Gandra et al., 2012; Claflin et al., 2015). These measures have lead to insight into the role of NAD(P) redox in oxidative stress (Ying, 2008; Murphy, 2009; Aon et al., 2010; Massudi et al., 2012a), as an index of oxygenation states (Barlow and Chance, 1976; Mayevsky and Rogatsky, 2007), and as a probe of mitochondrial (dys)function in age and disease (Heikal, 2010; Massudi et al., 2012b; Claflin et al., 2015). However, optical measures are typically limited to isolated tissues and are limited in depth penetration *in vivo*. A non-invasive measure of NAD(P) *in vivo* would open a window on cell and mitochondrial metabolism in age and disease as well as the impact of treatments to reverse metabolic dysfunction.

Non-invasive magnetic resonance methods now make detecting NAD(P)^+^ and NAD(P)H *in vivo* possible both in animals and humans (de Graaf and Behar, 2014; Zhu et al., 2015). A distinct spectral position (chemical shift, CS) in the phosphorus spectrum (^31^P MRS) is found for each metabolite representing the NAD(P)^+^ and NAD(P)H redox states (Lu et al., 2014). It may also be possible to distinguish the cellular location of these NAD(P) redox states since several metabolites are reported to show distinct CS in the ^31^P MR spectrum between these two compartments (Garlick et al., 1983; Hutson et al., 1989, 1992). For example, both ATP and NADP^+^ show striking differences in CS with Mg^2+^ binding at the low metal concentration in the cytosol vs. the 10-fold higher level found in mitochondria (Mas and Colman, 1984; Gout et al., 2014). Inorganic phosphate also shows a significant chemical shift *in vivo* as a result of the pH difference in cytosol vs. mitochondria (Garlick et al., 1983; Hutson et al., 1992; Kan et al., 2010). There are also promising studies suggesting that NAD(P) redox states can be distinguished between the mitochondria vs. the cytosol. These studies found that phosphoryl compounds related to NAD(P) showed CS differences between erythrocytes vs. the bathing solution that are large enough to be detected by ^31^P MRS (Kirk and Kuchel, 1988a,c). Thus, it may be possible to detect a NAD(P)H CS difference inside mitochondria relative to the cytosol in the ^31^P MR spectrum thereby providing a natural indicator of key player in oxidative phosphorylation.

Here we describe a unique resonance in the ^31^P MRS spectrum that conforms with the properties of NAD(P)H in mitochondria in human muscle *in vivo*. The aim of this study was to test the nature of this new resonance and determine if it provides a natural indicator of mitochondrial oxidative function. The first test compared this unique resonance to chemical standards of metabolites known to be present in the same region of the ^31^P MR spectrum, including NAD^+^, NADP^+^, NADH, and NADPH (Kushmerick et al., 1986). No overlap in the spectral position (chemical shift) of the unique resonance vs. that of known metabolites was found. Two additional tests were performed to determine whether dynamics elicited in this peak by exercise conformed with changes expected for mitochondrial NAD(P)H. This functional test was designed to elevate oxidative phosphorylation and involved 40 elderly subjects before and after exercise training. The pre-training test revealed a reciprocal decline in the unique resonance vs. elevation in NAD(P)^+^, which are changes consistent with oxidation of mitochondrial NAD(P)H to NAD(P)^+^. The post-training test found a greater change in the unique resonance after exercise training, which is consistent with elevated oxidation and the greater phosphorylation rate reported in these subjects. Taken together, these results provide evidence that this MRS detectable resonance conforms to the properties of mitochondrial NAD(P)H. Thus, this unique resonance holds promise as the first natural indicator of the inner workings of oxidative phosphorylation in the ^31^P MRS spectrum. It also holds promise as a sensitive indicator of the mitochondrial response to treatments designed to improve mitochondrial function.

## Methods

### Subjects

An elderly group consisted of 40 subjects (18 male, 22 female) ranging in age from 65 to 80 years (68.8 ± 5.9 years, means ± S.E.M.). Subjects were not involved in formal exercise training, were in good health and had no significant cardiac, neurological, or musculoskeletal disease, as we have described (Conley et al., 2000; Jubrias et al., 2001). All subjects voluntarily gave informed, written consent as approved by the University of Washington Human Subjects Review Committee and in accordance with the Declaration of Helsinki.

### MR methods

A General Electric 1.5 T Signa imager/spectrometer was used for the *in vivo* spectra as described (Conley et al., 2000). A 9 cm diameter surface coil tuned to the phosphorus frequency (25·9 MHz) was placed over the vastus lateralis muscle of the thigh. The B_1_ field homogeneity was optimized by off resonance proton shimming on the muscle water peak. The unfiltered PCr linewidth (full width at half-maximal height) was typically 4–8. Each subject had a high resolution ^31^P MR spectrum of the resting vastus lateralis muscle taken under conditions of fully relaxed nuclear spins (16 free-induction decays (FID) with a 16 s interpulse delay) using a spectral width of ±1250 Hz and 2048 data points. Since volume selective methods were not used in collection, the spectra represent signal from a hemispherical volume defined by the coil radius. No volume selective methods were used to collect the spectra. Dynamic changes during stimulation and recovery were made using a standard 1-pulse experiment with partially saturated nuclear spins (1.5 s interpulse delay). Artifacts due to movement were reduced by stabilizing the limb during the muscle contractions. All fully relaxed spectra were zero filled from 2048 to 4096 points, Fourier transformed with 15 Hz apodization, baseline corrected, and manually phased using the Mnova software package (Mestrelab Research, Santiago de Compostela (Spain)). No other spectral treatment was employed.

### Chemical phantoms

Metabolites that resonate in the NAD(P) region of the ^31^P MR spectrum were identified from muscle extracts (Kushmerick et al., 1986). Solutions containing these chemicals were prepared using binding constants taken from the USA National Institute of Standards and Technology (NIST) Critically Selected Stability Constants of Metal Complexes Database (see Kushmerick, 1997). All solutions contained (in mmol/L): EGTA 15, MOPS 80, free Mg^2+^ 1, Na^+^ 83, and K^+^ 52. The following were varied in individual solutions: ATP, ADP, P_i_, Creatine Phosphate (PCr), ADP, NAD^+^, NADH, NADP^+^, or UDP-Glucose. The ionic strength was maintained at 0.175 M, pH = 7.0 (36°C) in all solutions. High-resolution MR spectra of individual solutions in an NMR tube were taken at 4.7T (sweep width = 10,000 Hz, 16 K complex points, 128 FIDs, 5 s delay between pulses).

### Peak simulations

Resonances were simulated at 1.5T using the spin parameters and chemical shifts for the simulations determined from NAD^+^, NADP^+^, and NADH in physiological solution at 4.7T. The Spin Simulation feature of the Mnova software (Mestrelab Research, Santiago de Compostela (Spain)) permitted determining the spectral properties of these resonances. The line-width of the simulated resonances were fixed to that of α-ATP due to the similar chemical environment of the two phosphate groups in NAD(P). The details of this process have been described (Lu et al., 2014).

### NAD(P) region *In vivo*

**Figure 1:**
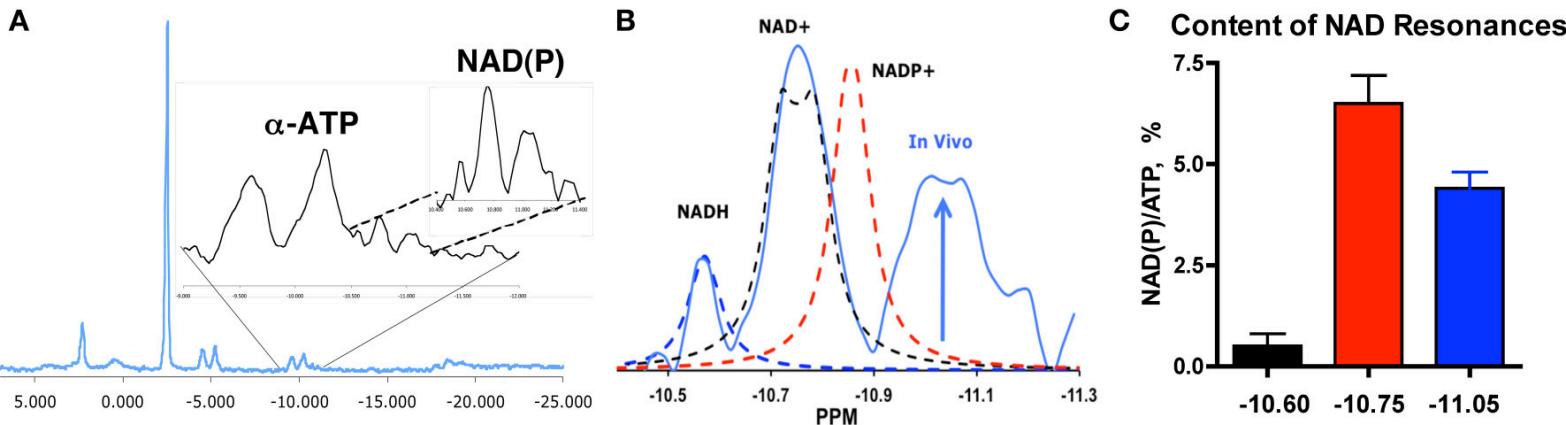
**NAD(P) resonances ***in vivo*** in human muscle**. **(A)** ^31^P magnetic resonance spectrum from human vastus lateralis at 1.5T with expansion of α–ATP and NAD(P). **(B)** Resonances for NADH, NAD^+^, and NADP^+^ (dashed lines) vs. the *in vivo* spectrum (blue) reveal an unidentified peak (arrow, −11.05 ppm). **(C)** Content of the three resonances *in vivo* [NAD(P)] per ATP content, mean % ± SEM, *n* = 39 (Conley et al., 2000).

The −10.4 to −11.3 region containing NAD^+^ and NADP^+^ was extracted from the fully relaxed spectra by fitting a Lorenzian line shape to the α–ATP peak (Figure 1). The spectral intensity of this region was integrated as well as three sub-regions: −10.5 to −10.68 ppm (NADH), −10.69 to −10.92 (NAD(P)^+^) and −10.93 to −11.30 (hereafter, −11.05 ppm). The dynamic changes in these regions were determined by subtraction spectra normalized to the α–ATP level between stimulated vs. resting muscle and during recovery vs. resting muscle.

### Stimulation and recovery protocol

The quadriceps muscles were activated by transcutaneous electrical stimulation of the femoral nerve, as previously described (Blei et al., 1993; Conley et al., 2000). Spectra were collected during rest, stimulation and recovery to measure the PCr, P_i_, ATP, and the NAD(P) region peaks. Spectra were averaged during each of the three periods to analyze the NAD(P) region peaks:

*Control period (60 s, 10 spectra):* Baseline data were obtained during resting muscle conditions to establish initial metabolite peak levels.
*Stimulation period (120 s, 20 spectra):* A 3 Hz electrical stimulation period was used to decrease [PCr] and activate NAD(P)H synthesis.
*Aerobic recovery (300 s, 50 spectra):* upon cessation of stimulation, PCr recovery was followed until restoration of baseline levels during which NAD(P)H oxidation was determined.

### Statistics

Pre- vs. post-training differences relative to zero were tested with a 2-tailed Student's *t*-test. No adjustment for multiple comparisons was made as per reference (Perneger, 1998). Statistical differences are reported at *P* < 0.05. Means are reported ±SEM.

## Results

Here we show evidence for a unique resonance in the ^31^P spectrum corresponding to NAD(P)H in mitochondria. To identify this resonance, we first compare the unique resonance against known metabolites in muscle. Second, we test the link between the unique resonance vs. the change in mitochondrial content that came with exercise training in these subjects. Third, we use an acute exercise bout to activate NAD(P)H oxidation to test for changes in the resonance and determine whether these changes (oxidation) parallel the improvement in phosphorylation found after exercise training in these subjects.

### Unique resonance in ^31^P spectrum *In vivo*

The presence of an unidentified peak in the ^31^P MR spectrum of human muscle is illustrated in Figure 1. An expansion of the spectrum in the α–ATP region shows the NAD(P) peak region from −10.4 to −11.3 ppm. This spectrum was taken from an elderly vastus lateralis muscle under MR conditions in which peak areas reflect their chemical content (i.e., fully relaxed conditions). The resonances for chemical standards of NADH (−10.6 ppm), NAD^+^ (−10.75 ppm), and NADP^+^ (−10.84 ppm) are superimposed on the NAD region from the *in vivo* spectrum in Panel B [UDP-glucose is also present (Kushmerick et al., 1986) and resonates in this region at −10.84 in *in vitro* solutions that emulate *in vivo* conditions]. This comparison highlights the presence of an additional resonance centered at −11.05 ppm in the NAD(P) region. Thus, the unique resonance at −11.05 ppm does not correspond with resonances for metabolites in this spectral region that have been identified in tissue extracts (Kushmerick et al., 1986).

### Unique resonance is 40% of total NAD(P) integral

The NAD(P) integral is 11% of the area of α–ATP (11.5 ± 1.0%), which agrees with the total NAD per ATP content (7–12%; Sahlin, 1983; Henriksson et al., 1986) reported from biochemical analyses in human vastus lateralis muscle (Sahlin, 1983; Henriksson et al., 1986; Ren et al., 1988). Based on the ATP level in these muscles (5.8 mM; Conley et al., 2000), the full integral (0.7 mM) contains 0.4 mM NAD(P) (60% of the integral) vs. 0.5 mM measured for total NAD in young muscle (Henriksson et al., 1986). The unique resonance at −11.05 ppm is the second largest peak in the NAD(P) integral (Figure 1C), which translates to 0.25 mM (38% of the total). This is close to the value (0.26 mM) estimated from the NADH content of muscle mitochondria (heart, 10 nmole/mg mitochondrial protein, Alano et al., 2007) and the mitochondrial volume density of the elderly muscle in this study [3% (Conley et al., 2000), i.e., 10 nmole/mg × mg protein/μl (Vinnakota and Bassingthwaighte, 2004) × 0.03 μl mito/μl muscle = 0.26 nmole/μl or 0.26 mM]. Thus, two key segments of the NAD(P) region *in vivo* are consistent in size with the contents of total NAD^+^ assayed in muscle and NADH estimated from mitochondrial volume density of human muscle.

In contrast, the small resonance at −10.6 ppm associated with NADH in solution was 5% of the total integral in the NAD(P) region or ~0.01 mM. A low NADH content in the cell is expected from the oxidized NAD redox state maintained by enzyme equilibria linked to the phosphorylation potential (Williamson et al., 1967; Stubbs et al., 1972). This low value agrees with the NADH levels in older bioluminescent assays on human vastus lateralis tissue (0.02 mM at rest, Henriksson et al., 1986). These findings suggest that the chemical standard for NADH at −10.6 ppm identifies the resonance for the small cellular NADH pool, while the larger −11.05 ppm resonance is more consistent with the mitochondrial NAD(P)H pool. Thus, the metabolite content represented by the −10.4 ppm to −11.3 ppm integral, which includes the unique resonance at −11.05 ppm, is in reasonable agreement with the total NAD(P) content reported from biochemical analysis of vastus lateralis muscle tissue.

### Increase in −11.05 ppm peak and mitochondrial volume with exercise training

**Figure 2:**
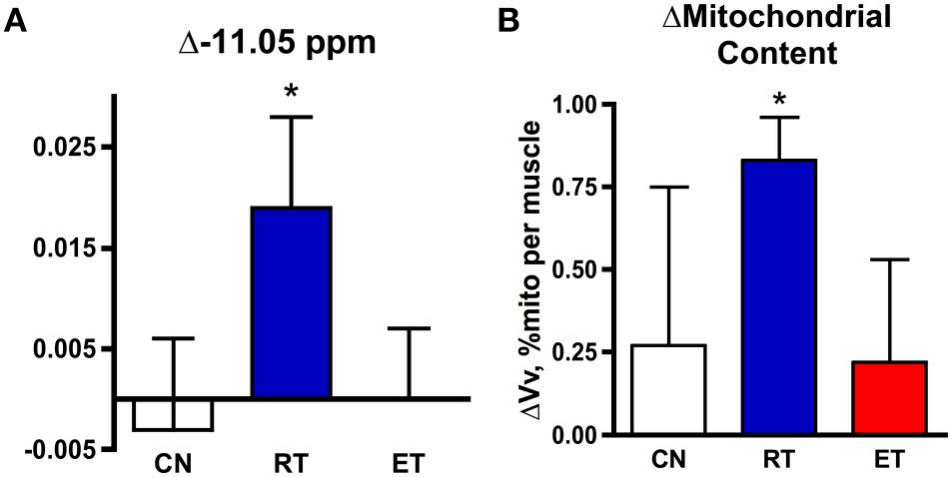
**Changes in NAD(P) resonances and mitochondrial volume in control (CN), resistance trained (RT, blue), and endurance trained (ET, red) groups**. Change with exercise training in **(A)** the −11.05 ppm peak area, and **(B)** mitochondrial volume density. ^*^−*P* < 0.05 post- vs. pre-training.

Our second test of the nature of the unique resonance took advantage of the increased mitochondrial content that accompanied a 6-mo exercise training program in these subjects (Jubrias et al., 2001). We tested whether the −11.05 ppm resonance increased in proportion to the elevation in mitochondrial volume density found with resistance training (RT) in these subjects (Δ31%). Figure 2 shows the significant changes in the −11.05 ppm resonance with RT in elderly subjects (Δ39%), while no change was found on average in either property in control (CN) or endurance trained (ET) groups. Also, no change was found in the NADH peak area at −10.60 ppm in the RT group (0.1 ± 0.5% ATP). Thus, the adaptation in the −11.05 ppm peak was exclusively associated with that of mitochondrial volume density with exercise training.

### Reciprocal change in −11.05 ppm peak vs. NAD(P)^+^ with elevated oxidation

**Figure 3:**
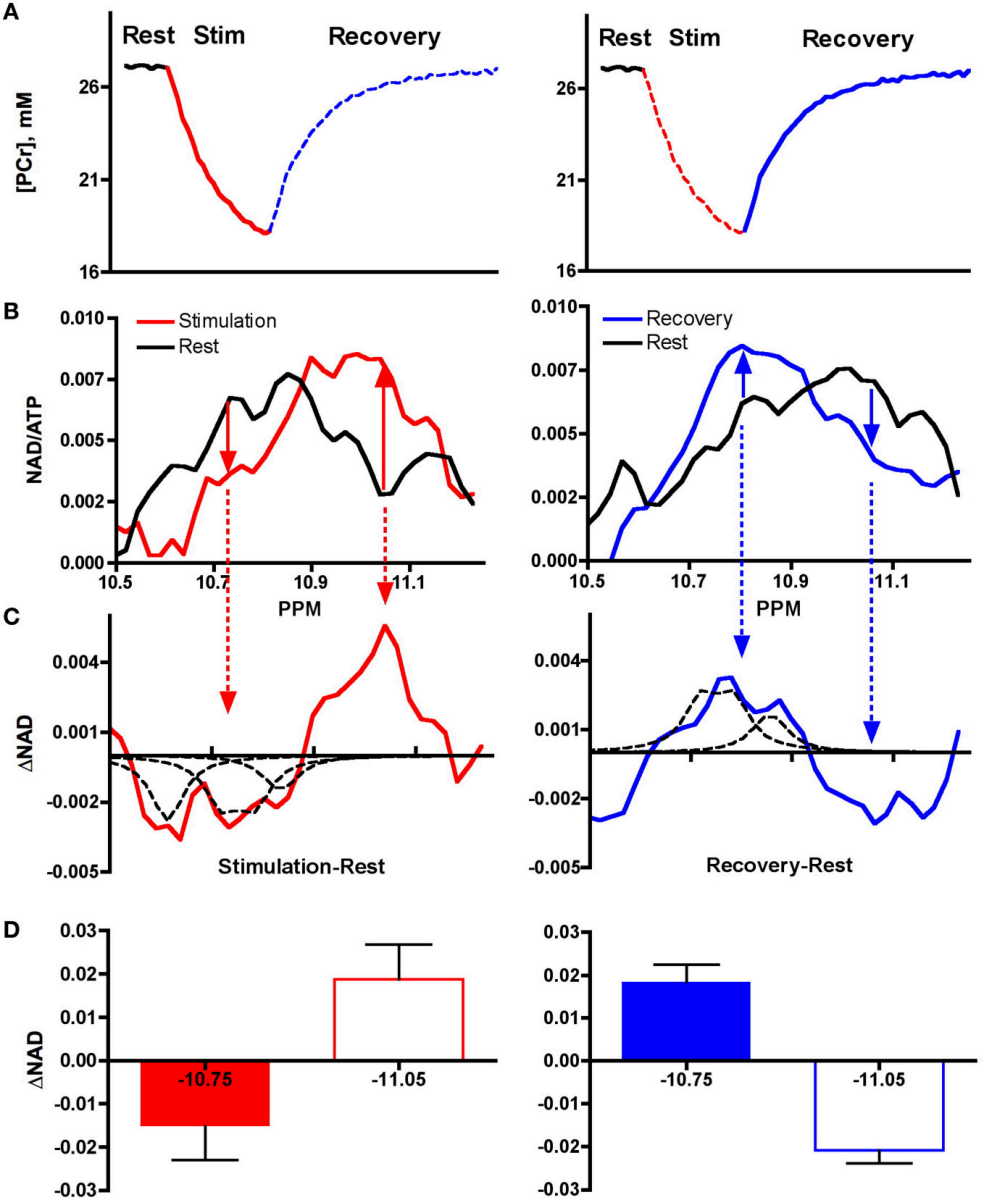
**Spectral changes during stimulation and recovery from exercise**. **(A)** Exercise elicits elevated ATP use (PCr decline, red line) and oxidative phosphorylation (PCr re-synthesis, blue line). **(B)** Altered ^31^P Spectra in stimulation (red) and recovery from exercise (blue) vs. resting muscle (black). **(C)** Subtraction of spectra region in resting (black) vs. stimulated muscle (red) and vs. recovering (blue) muscle reveals reciprocal changes in −10.75 ppm and in −11.05 ppm regions. The dashed peaks represent the resonances for the chemical metabolites in solution: NADH (−10.6 ppm), NAD^+^ (−10.75 ppm), and NADP^+^ (10.83 ppm). **(D)** Reciprocal changes in integrated areas of −10.75 ppm and −11.05 ppm regions.

A third test involved an exercise and recovery bout designed to alter the NAD(P) resonances by elevating oxidative phosphorylation, as shown in Figure 3.

Figure 3A shows the changes in creatine phosphate (PCr), a natural indicator of cell ATP use and re-synthesis, during an exercise and recovery bout. Exercise consisted of a 2 min muscle stimulation period that increased ATP use resulting in depletion of PCr level from the resting state (red line). The curvilinear shape of the PCr breakdown curve indicates that activation of ATP synthesis by mitochondria reduces the net breakdown toward the end of the stimulation period. Once stimulation ends and contractile ATP demand ceases, oxidative phosphorylation predominates (blue line) and PCr is resynthesized to quickly return to resting levels.

Figure 3B shows the ^31^P spectra of the NAD(P) region taken from 10 subjects during these two periods. The spectrum collected from resting muscle (black line) represents the low oxidative phosphorylation condition. The spectrum collected from muscle during stimulation is shown in by red lines (left hand panels) and that collected during the recovery period of elevated oxidative phosphorylation is shown by the blue lines (right hand panels). The solid arrows show the reciprocal changes in the −10.75 ppm and in the −11.05 ppm region.

Figure 3C shows the net differences in these regions with subtraction of these spectra. The change in the −10.75 ppm region is outlined by several dashed peaks. These peaks represent the resonances of the following chemical metabolites in solution: NADH (−10.6 ppm), NAD^+^ (−10.75 ppm), and NADP^+^ (10.83 ppm). Note that the decline in the −10.75 ppm region with stimulation (red arrows) is matched by an equal rise in the −11.05 ppm region. The opposite changes are seen during recovery from stimulation (blue line): the −11.75 ppm region rises and the −11.05 ppm region declines.

Figure 3D shows the integration of the peak areas in the difference spectra. Equal and opposite change in the −10.75 vs. −11.05 ppm region is confirmed by no significant difference in a paired *t*-test of the absolute integrated areas of the two regions in stimulation or recovery (*P* > 0.15 each). Such reciprocal changes are expected for a trade-off of oxidized and reduced forms of NAD(P)^+^. The correspondence of the dashed peaks representing the resonances for NAD^+^ and NADP^+^ and the change in the −10.75 ppm region is strong support that this region represents net changes in NAD(P)^+^. Similarly, the reciprocal change in the −11.05 ppm region suggests that change in NAD(P)H occurs in the region of the unique resonance. These changes are consistent with the dynamics expected for reduction and oxidation of mitochondrial NAD(P)H. Taken together, these results point to mitochondrial NAD(P)H dynamics occurring at the downfield spectral position (−11.05 ppm) from the resonance defined by the chemical NADH (−10.6 ppm) that likely defines the cell NADH.

### Exercise training accelerates NAD(P) dynamics and phosphorylation

Our final test was whether the −11.06 ppm peak changes with an independent measure of oxidative phosphorylation. These subjects showed increased phosphorylation capacity after endurance (ET) and resistance (RT) training (Jubrias et al., 2001). This capacity was measured during the stimulation-recovery experiment shown in Figure 3, which was undertaken in each subject and repeated after exercise training. Table 1 shows the changes in the −11.05 ppm peak in the three groups in this training experiment and reveals that both ET and RT groups show a net decline in the −11.05 ppm peak after training. No change is apparent in the control group. A corresponding net rise that balances the decline in the −11.05 ppm peak area is apparent in the −10.6 ppm peak (ET) or the −11.75 ppm peak (RT) with training. This rise in the integrated peaks in the −10.5 to −10.9 ppm region vs. the −11.05 ppm region demonstrates a net stoichiometry in the changes of the NAD(P) peaks with training. This net decline in the −11.05 ppm region during elevated oxidation following training further supports this spectral region as representing the mitochondrial metabolites, NAD(P)H.

**Table 1:** **Change in peak areas during the exercise recovery period in post- vs. pre-trained muscle**.

**Group**	**Δ−10.60 ppm**	**Δ−10.75 ppm**	**Δ−11.05 ppm**
CN	2.3 ± 6.3	−1.0 ± 0.5	−0.2 ± 1.3
RT	0.3 ± 0.7	1.5 ± 0.4^*^	−1.7 ± 0.7^*^
ET	1.5 ± 0.6^*^	0.1 ± 1.4	−1.7 ± 0.6^*^

*Units are % NAD per α–ATP peak for three resonances at distinct chemical shifts (ppm) in the NADP region. Values are means ± SE. Δ defines post- vs. pre-training changes in the integral of area of each resonance per integral of the α–ATP resonance*.

*The groups are: CN, control; RT, resistance training; ET, endurance training groups*.

*Δ−10.6, Δ−10.75, and Δ−11.05 are the chemical shifts (ppm) for the resonances defined by external standards for the reduced (NADH) and oxidized (NAD(P)^+^) forms and a third resonance not identified by external standards, respectively*.

* *Bold font, P < 0.05 post- vs. pre-training*.

**Figure 4:**
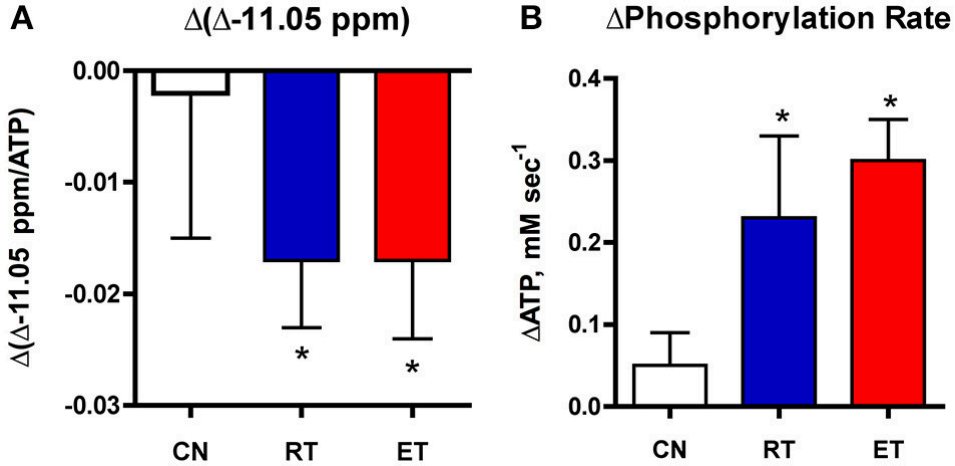
**Changes in the −11.05 ppm peak (A) and phosphorylation (B) with exercise training**. Net decrease in the −11.05 peak and net elevation in oxidative phosphorylation during recovery is shown in muscle after resistance (RT) and endurance (ET) training vs. controls (CN). ^*^P < 0.05.

Figure 4 shows that the significant decline in the −11.05 ppm region (−10.9 to −11.3 ppm) mirrored the net increase in phosphorylation with training in these subjects. These reciprocal changes suggest that the Δ-11.05 ppm region reflects the greater oxidation of NAD(P)H that is expected with the elevated phosphorylation (ΔPCr) found after training. Together, these results support that mitochondrial NADH oxidation *in vivo* is represented by the decline in the unique resonance at −11.05 ppm in the NADP region. Further, these results indicate that the −11.05 ppm peak is a sensitive indicator of the impact of an intervention on improving mitochondrial oxidation in elderly muscle.

## Discussion

**Figure 5:**
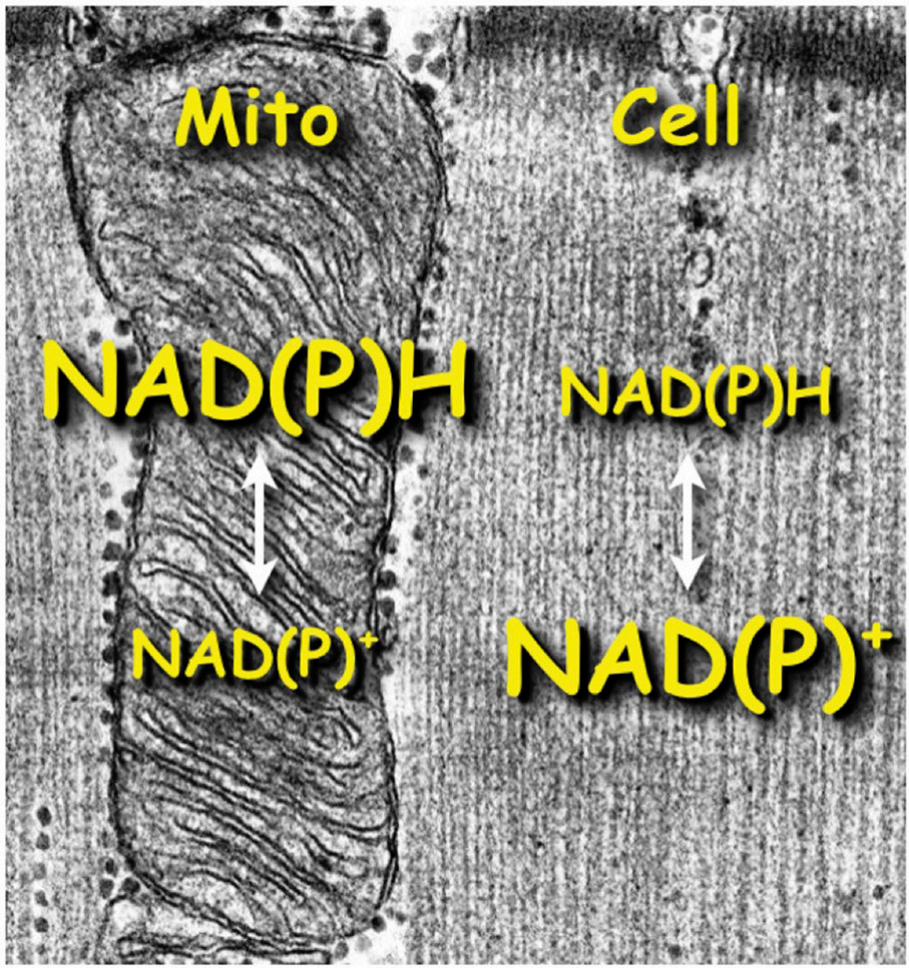
**Scheme illustrating the disparity of NAD(P) redox states expected in the cytosolic vs. mitochondrial compartments**.

The metabolic role and redox state of the NAD(P) metabolites depend on their cellular location as illustrated in Figure 5. NADH is a critical substrate for oxidative phosphorylation and predominates over NAD^+^ in mitochondria. The opposite is true for the oxidized cytosol in which enzyme equilibria linked to the phosphorylation potential holds NAD(P)H content low and NAD(P)^+^ high (Williamson et al., 1967; Stubbs et al., 1972). Here we describe a unique resonance at −11.05 ppm in the *in vivo* ^31^P MR spectrum that conforms with the properties of mitochondrial NAD(P)H. This unique resonance has the high chemical content and the dynamic changes with oxidative phosphorylation that are expected for the NAD(P) redox state in mitochondria. In contrast, a second peak identified by an NADH chemical standard (at −10.6 ppm) is consistent with the low metabolite content expected for cytosolic NADH. Three results summarize the evidence that the −11.05 ppm peak is a natural indicator of the content and dynamics of mitochondrial NAD(P)H and thereby provides a intrinsic probe of oxidative phosphorylation *in vivo*:

- Quantitative agreement: A correspondence is found between the chemical content represented by the −11.05 ppm peak and the NAD(P)H level estimated from the mitochondrial content of these muscles and their changes with exercise training.
- Reciprocal dynamics with NAD(P)^+^: The equal and opposite change in the −11.05 ppm peak vs. NAD^+^ with exercise and recovery reflects the expected trade-off between reduced (NAD(P)H) and oxidized (NAD(P)^+^) redox states in mitochondria.
- Dynamics parallel phosphorylation: A net decline in the −11.05 ppm peak mirrors the greater phosphorylation after exercise training, which supports this unique resonance as a sensitive natural indicator of changes in mitochondrial NAD(P)H oxidation with an intervention.

### NAD(P) *In vivo* vs. *In vitro*

Several lines of evidence indicate good agreement of the NAD(P) metabolite levels determined in the ^31^P MR spectrum *in vivo* and those reported in isolated human muscle by *in vitro* methods. The metabolite content represented by the peaks in the NAD(P) region (−10.5 to −11.3 ppm; 11% NAD/ATP) is in agreement with biochemical assays of NAD in human vastus lateralis (Sahlin, 1983; Henriksson et al., 1986). Chemical identification of the peaks in this region reveals that NAD(P)^+^ accounts for 60% of the total peak area while only 5% of the total is accounted for by the chemically defined NADH (−10.6 ppm). The chemical content of the NAD(P)^+^ region (0.4 mM) is consistent with estimates for NAD^+^ derived from biochemical assays in young muscle (0.5 mM). The remaining 38% of the peak area of the NAD(P) region is represented by a peak centered at −11.05 ppm. Both the MR determination and that derived from independent measures estimate a NAD(P)H content of ~0.26 mM.

### Two resonances reflect NAD(P)H *In vivo*

A large difference in peak areas is expected from *in vitro* results if cytosolic vs. mitochondrial NAD(P)H can be distinguished in the ^31^P MR spectrum (Williamson et al., 1967; Stubbs et al., 1972). The chemical content represented by the −10.60 ppm peak was low (5% of NAD(P) integral) relative to the unique resonance at −11.05 ppm (38%) as expected for cytosolic NADH, which is maintained at a low level by enzyme equilibria linked to the phosphorylation potential (Williamson et al., 1967; Stubbs et al., 1972). The larger chemical content of the −11.05 ppm corresponds with the predominance of mitochondrial NAD(P)H pool. This agreement of the size of two ^31^P MR resonances with the expected NAD(P)H contents provides evidence that the cytosolic vs. mitochondrial NADH pools can be distinguished by MRS *in vivo* (Krebs and Veech, 1969; Stubbs et al., 1972).

### Compartmentalization of NAD(P)H

The basis of the distinct spectral positions of NAD(P) is likely due to the changes in physical properties when the metabolite is located inside a compartment, such as in mitochondria. For example, enhanced fluorescence efficiency occurs with NAD(P)H inside mitochondria due to binding to complex I of the electron transport chain (Blinova et al., 2008), which permits highlighting mitochondrial NAD(P)H by optical spectroscopy and imaging. A second example is the different chemical shift in the ^31^P MR spectrum seen with phosphoryl compounds located inside erythrocytes (Kirk and Kuchel, 1988a,c; Larkin et al., 2007). Compounds related to NAD(P) show a Δ−0.5 ppm CS in the red blood cell, which is similar to the shift of the −11.05 ppm vs. −10.6 ppm NAD(P)H peak *in vivo* in muscle (Kirk and Kuchel, 1988a,c; Larkin et al., 2007). The mechanism for this downfield CS in red cells is reported to be elevated hydrogen bonding resulting from the high protein concentration of the erythrocyte (predominantly hemoglobin content) (Kirk and Kuchel, 1988b; Larkin et al., 2007). A high protein content is also characteristic of mitochondria (Vinnakota and Bassingthwaighte, 2004). Thus, the segregation of phosphoryl compounds inside a high protein environment provides a physical mechanism for the downfield CS of the −11.05 ppm peak (reflecting NAD(P)H in mitochondria) relative to NAD(P)H in the cell (−10.6 ppm) *in vivo*.

### Increase in −11.05 ppm peak and mitochondrial content with exercise training

A test of the identity of the −11.05 ppm peak is provided by the changes in the peaks of the NAD(P) region and in mitochondrial properties with exercise training (Figure 2). A shift in NAD(P) metabolite levels in correspondence with changes in mitochondria have been reported in several tissues with calorie restriction in mice in *in vitro* assays (Chen et al., 2008). One mechanism for an NAD^+^ increase is a rise in the enzyme responsible for NAD^+^ biosynthesis—NAMPT—that accompanies calorie restriction and exercise training (Costford et al., 2009). These adaptations in NADH and mitochondria were not uniform across tissues in calorie restricted mice (Chen et al., 2008). They also differed between training modes in the human muscle (Figure 2). A higher −11.05 ppm peak (Δ39%) was found in proportion to the greater mitochondrial content with RT (Δ31%), but neither property changed significantly with ET (Figure 2). In contrast to the −11.05 ppm peak, the chemically defined NADH peak at −10.6 ppm did not change despite the increased mitochondrial content in RT. This stability of the peak at −10.6 ppm in the face of a large increase in mitochondrial content is consistent with this resonance representing NAD(P)H in the cytosol. In contrast, a mitochondrial origin for the −11.05 ppm peak is supported by the unique rise of this resonance with mitochondrial volume density after exercise training.

### Activating NAD(P)H generation and oxidation

A functional test of the identity of the −11.05 ppm peak is provided by the dynamics of the peaks in the NADP region with exercise and recovery. These dynamics are elicited by a protocol that activates increased energy use (as shown by PCr depletion from the resting state) followed by a period of elevated oxidative phosphorylation after exercise (as shown by restoration of PCr, Figure 3A). The peaks in the NAD region change in concert with these PCr dynamics during exercise and recovery (Figure 3B) to reveal the trade-offs in the reduced vs. oxidized redox states of NAD(P) (Figure 3C) (Jobsis and Duffield, 1967; Wendt and Chapman, 1976). Such trade-offs are evident from the changes in the −11.05 ppm peak, which are matched by equal and opposite changes in oxidized NAD(P) (Figure 3D). Similar changes are apparent in optical fluorescence studies that indicate reduced mitochondrial NAD(P)H with muscle stimulation and oxidized NAD(P)H during elevated oxidative phosphorylation in recovery (Jobsis and Duffield, 1967; Wendt and Chapman, 1976; Gandra et al., 2012; Claflin et al., 2015). A rise in NAD(P)H generation under intense exercise has also been found in glycolytic type II fibers of human muscle in biochemical studies (Ren et al., 1988), while reduced NADH is found during sustained exercise in the oxidative, type I fibers of humans (Ren et al., 1988). Thus, the functional dynamics of the −11.05 ppm peak parallel the changes in mitochondrial NAD(P)H during exercise cycles reported in independent studies. These reciprocal changes in spectral regions are consistent with the −11.05 ppm peak representing mitochondrial NAD(P)H.

### Sensitive index of mitochondrial oxidative improvements

An *in vivo* measure of mitochondrial NAD(P)H holds the promise of providing a natural indicator of oxidation adaptations with an intervention in human muscle. A test of the −11.05 ppm peak as such an indicator is provided by the results of exercise training that raised oxidative phosphorylation in these subjects (Jubrias et al., 2001). Figure 4 shows a net drop in the −11.05 ppm resonance in an exercise bout after exercise training. This net decline parallels the net increase in phosphorylation rate and suggests that the −11.05 ppm peak reflects increased oxidation of mitochondrial NADH in the trained subjects. Independent findings of faster oxygen uptake after exercise training support these results. Human muscle O_2_ uptake during recovery from exercise is increased and the kinetics of whole-body oxygen uptake is more rapid at the onset of exercise after training in adult and elderly subjects (Zoladz et al., 2006; Murias et al., 2010). The alternative is a higher ATP flux without increased O_2_ uptake as a result of improvement in the coupling of generating ATP per O_2_, which also occurs with exercise training (Conley et al., 2013). A recent analysis concluded that both mechanisms are activated: greater electron transport chain flux and more efficient generation of ATP appeared to contribute nearly equally to the phosphorylation improvements in these subjects (Conley, 2016). The results in Figure 4 support this independent analysis to demonstrate that elevated oxidation is a part of the improvement in mitochondrial capacity to generate ATP after training. These results indicate that the dynamics of the −11.05 ppm peak captures the mitochondrial oxidation improvements that underlie greater phosphorylation. Importantly, both the oxidation and phosphorylation measures come from the same ^31^P MR spectra collected in a single exercise test in human muscle *in vivo*.

### Limitations and future directions

The low chemical content of NAD(P) related metabolites (<1 mM) *in vivo* is a limitation to detecting this natural indicator by ^31^P MRS. Measurements are possible at the low field strength of 1.5T because of the trade-off with high B_1_ field homogeneity at 1.5T, which provides good resolution of the individual NAD(P) peaks despite the low signal-to-noise. The large diameter coil (9 cm) used in this study and large muscle mass of the quadriceps group (~1 kg) also helped to improve the signal for detecting these low concentration metabolites. Nonetheless, the high standard errors on some of the determinations may be due to this combination of low chemical content and low MRI field strength. However, two advances allow improved signal-to-noise over that found in the study. The first advance is the higher field strength (3T) MRIs that are now available in most medical centers that focus on human metabolic research. A second advance is the ability to decouple protons from phosphorus to increase the signal-to-noise of the NAD(P) peaks. Decoupling is a method derived from high-resolution MR spectroscopy that is now a standard feature of 3T MRIs with multi-nuclear detection (e.g., ^31^P MRS) capability. Thus, the availability of the higher 3T field strength for human studies and the ability to further enhance signal-to-noise with proton decoupling will make detecting of the NAD(P) peaks and their dynamic changes with an intervention possible for clinical research studies.

## Summary

Here we show that a unique resonance in the *in vivo* ^31^P spectrum provides a natural indicator of mitochondrial oxidation and its improvement with treatment. This assignment comes from the known compartmentalization of NADH between the highly oxidized cell (−10.6 ppm) vs. more reduced mitochondria (−11.05 ppm). A functional test that followed NAD(P) dynamics during exercise and recovery cycles confirmed that the −11.05 ppm peak reflected oxidation and reduction of mitochondrial NAD(P)H. Finally, the greater −11.05 ppm peak changes in the exercise test paralleled the faster phosphorylation rate in these subjects after exercise training. This correspondence provides further support that this unique resonance is a natural indicator of mitochondrial oxidation. Thus, intrinsic probes are present in the ^31^P MR spectrum that reveal the NAD(P) redox disparity between mitochondria and cell and provide a natural indicator of oxidative phosphorylation that is sensitive to improvements in mitochondrial function with treatment.

## Author contributions

KC, SJ, and ES conceived and designed the study; all authors contributed to the collection, analysis, and interpretation of data; KC and ES were involved in drafting or revising the paper.

## Funding

This work was supported by NIH grants RC2AG036606, R01AR41928, R01AG10853, and a Nathan Shock Center Pilot Project award, as well as Seattle Children's Mitochondrial Guild and the Royalty Research Fund of the University of Washington.

### Conflict of interest statement

The authors declare that the research was conducted in the absence of any commercial or financial relationships that could be construed as a potential conflict of interest.
